# Nanoscale mesh acts as anti-adhesive surface against particulate contamination in eyes of whiteflies

**DOI:** 10.1038/s41598-024-69059-2

**Published:** 2024-08-06

**Authors:** Tomer Urca, Fritz-Olaf Lehmann, Elena V. Gorb, Stanislav N. Gorb

**Affiliations:** 1 Department of Animal Physiology, University of Rostock, Albert-Einstein Str. 3, 18059 Rostock, Germany; 2 Department Functional Morphology and Biomechanics, Zoological Institute of the University of Kiel, Am Botanischen Garten 1-9, 24118 Kiel, Germany

**Keywords:** Adhesion, Contact mechanics, Surfaces, Vision, *Trialeurodes vaporariorum*, *Bemisia tabaci*, Sternorrhyncha, Aleyrodidae, Biomechanics, Biomaterials, Entomology, Structural properties

## Abstract

In many insects the surface of the eye is nanostructured by arrays of protuberances termed ommatidial gratings which provide the cuticle with anti-reflective, anti-wetting and self-cleaning properties. The hypothesised anti-contamination role of the gratings against dust and pollen results from theoretical predictions on grating geometry and experiments on synthetic replicas of ommatidia surfaces but has not yet been proven in an animal. Whiteflies are biological test beds for anti-contamination surfaces because they deliberately distribute wax particles extruded from abdominal plates over their entire bodies. The numerous particles protect the animal against water evaporation and radiation, but may severely impair vision. Using scanning electron microscopy (SEM) and CryoSEM, we here show that the cornea of whiteflies exhibits ~ 220 nm wide mesh-like structures forming hexagonal gratings with thin ~ 40 nm connecting walls. Quantitative measurements of wax particles on the eye show that the nanostructures reduce particle contamination by more than ~ 96% compared to other areas of the cuticle. Altogether, our study is the first description of a predicted optimized grating geometry for anti-contamination in an arthropod. The findings serve as evidence of the high effectiveness of nanostructured surfaces for reducing contact area and thus adhesion forces between biological surfaces and contaminating particles.

## Introduction

The surface of insect compound eyes often yields nanoscale protuberances termed corneal nipples or ommatidial gratings^1–6^. A recent comprehensive investigation on 23 insect orders and some non-insect arthropods reports a large diversity of these corneal nanostructures^7^. They fall into four distinctive groups: (1) nipple-like nanostructures, (2) maze-like nanostructures, (3) parallel strands and ridges, and (4) novel dimple-type nanostructures, including various intermediate forms. These nanostructures resemble Turing patterns that may result from reaction–diffusion mechanisms during the development of the animal^7,8^. In general, nipple-like nanostructures are found in lepidopterans and dipterans as regularly and irregularly packed protrusions, respectively, or in Trichoptera, Mecoptera, Megaloptera, Hemiptera, Psocoptera, Thysanura, Raphidioptera, Neuroptera, Orthoptera, and Odonata as regularly packed, but irregularly shaped nipple-like protrusions. Maze-like nanostructures are found in Coleoptera, Trichoptera and Hymenoptera, as well as in some arachnids. Parallel strands/ridges are formed by fusion of nipple-type protrusions and described in dipterans and true spiders. Eventually, a more diverse group of novel dimple-type nanostructures has been found in Siphonaptera, Coleoptera, Hymenoptera, Hemiptera, Blattodea, Dermaptera, and in centipedes^7^.

It is generally accepted that nanoscale gratings establish an anti-reflective feature of the insect compound eye^9–12^. Biomimetic applications of nanoscale gratings in high-performance optics and coatings for solar batteries have demonstrated the effectiveness of these structures^13,14^. Depending on their contact mechanical properties and surface chemistry, ommatidia gratings also function as hydrophobic anti-wetting^15,16^ and self-cleaning surfaces^17^. Their effectiveness, though, depends on grating geometry and the surface energy of the contaminant. Measurements of adhesion using atomic force microscopy estimated the pull-off forces of different polymer mould copies of nanostructures from three insect groups (Odonata, Lepidoptera and Diptera). The latter measurements were compared with smooth control surfaces with same curvature radii as single ommatidia lacking the nanostructures^17^. The comparison revealed that the decrease in real contact area caused by corneal gratings greatly reduces adhesion to the ommatidia surface. This anti-adhesive property should prevent the accumulation of contaminants on the eye surface.

Maintaining a clean eye surface is crucial for vision-dependent behaviours in insects. Many groups of pollinating insects, such as bees and flies, as well as subterranean beetles, constantly interact with pollen and dust contamination that impairs eye sight and thus body posture reflexes, orientation and navigation^18^. For this reason, the eyes are actively cleaned by grooming^19,20^ as shown for various species of Hymenoptera^21^, Diptera^22^, Hemiptera^23^ and Coleoptera^24^. In addition to contaminants from the environment, some insect groups, such as whiteflies, actively produce own contaminants, covering their body with microscopic wax particles. It has been suggested that the wax particles protect the animal body against evaporation and provide a barrier for microorganisms^25^. As in the case of terrestrial higher plants, particulate waxes may provide additional waterproofing against transpiratory water loss and water influx by breaking up and shedding droplets falling on their surface^26–29^. This is similar to aphid waxes^26^ and brochosomes of leafhoppers^30,31^, which provide protection against the animal's own honeydew excrements. Furthermore the strong light scattering properties of the wax particles reflect visible solar radiation leading to the white colour of the whiteflies^25,32^. Waxes may also prevent adhesion of pathogenic fungi to the animal surface^33,34^. However, these wax particles may adhere to the surface of the compound eye, severely reducing the animal's visual capacity. The self-contamination with wax particles should thus have created an evolutionary pressure on efficient anti-contamination mechanisms of the cornea. Whiteflies thus serve as a test bed for studying anti-contamination hypotheses in a more natural context than it has been done before.

In this study, we show that whiteflies yield ommatidia gratings that effectively prevent the compound eyes from being covered with wax particles. We reconstruct the shape and quantify the geometry of both wax particles and the gratings in two species of whiteflies. Measurements of contamination of various cuticle areas allow us to estimate the efficiency with which particles are removed from the eyes. The obtained results are discussed in the light of recent studies in the field of contact mechanics, providing strong evidences for the high effectiveness of gratings in comparison to parabolic protrusions for the reduction of the contact area and thus adhesion forces between cuticle and contaminants.

## Results

**Figure 1:**
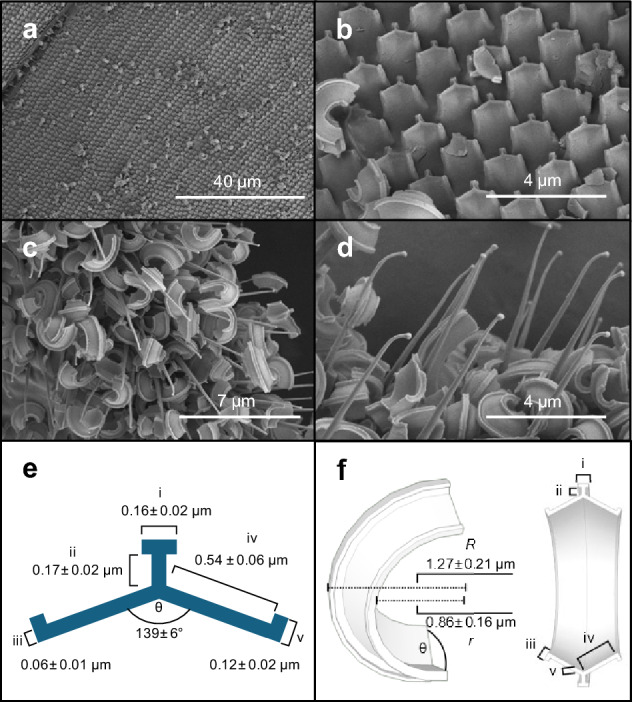
SEM images of wax producing and wax collecting sites in *Trialeurodes vaporariorum*. (**a**) Wax plate. (**b**) Rows of microtrichia arranged in groups of three on the surface of the wax plate. (**c**,**d**) Wing surface covered by acanthae collecting particles in multiple layers and mechanically fastening them to the surface. (**e**) The dimensions of the wax particles are defined by the dimensions of the templates from which they are extruded. (**f**) Reconstruction of the curved, three dimensional shape of wax particles after they are shaven off the abdominal plates. Data are means ± standard deviation (N = 18 measurements). Roman numerals indicate corresponding template and particle structures. The models in *e* and *f* are created with SketchUp® (Trimble Inc., Westminster, Colorado, U.S.).

The wax plate in *T. vaporariorum* consists of thousands of templates which appear as rows of microtrichia on the surface arranged in groups of three (Fig. 1a, b). The wax material is extruded to the surface through wax canals as three-armed continuous ribbons (Fig. 1e). The end of the ribbons are broken off into curved pasta-like particles with ~ 1.0 µm thickness and a radius of 1.27 ± 0.21 µm (N = 18 particles). They are produced by the movements of the tibiae of the hind legs that are drawn across the plates (Fig. 1f). The majority of the animal's cuticular surface, including the wings, is covered in ~ 6.28 ± 1.43 µm (N = 67) long conical-shaped cuticular outgrowths with a ~ 0.50 µm diameter base which terminate in ~ 0.11 µm diameter heads (Fig. 1c, d). These filiform nanostructures are likely acanthae (terminology after^35^). The mean distance of ~ 1.26 ± 0.28 µm (N = 71 measurements) between neighbouring acanthae is equal to the size of the wax particles (*t*-test, p > 0.05). Due to the size relationship and the small inner diameter of the wax particles (~ 0.86 ± 0.16 µm, Fig. 1f), the particles are caught and mechanically interlock to the acanthae-covered surface. The thickened head at the tip of the acanthae prevents the particles from slipping off of the acanthae. The cuticle surface may potentially collect up to six layers of wax particles and mechanically retain them during mechanical disturbances such as thorax vibration in flight, vibrational communication and mating. Even in animals that are dried in ethanol, an elevated number of wax particles remain on the cuticle due to the mechanical interlock.

**Figure 2:**
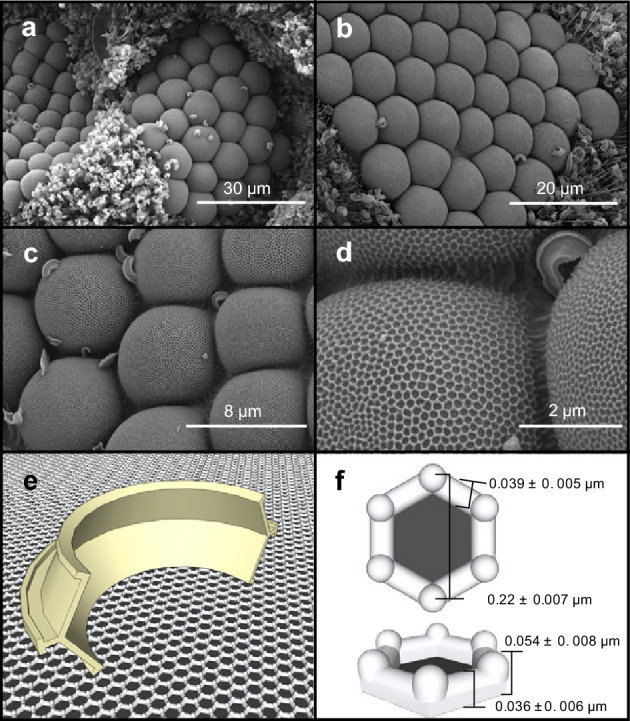
SEM images of the compound eyes of whiteflies showing ommatidia gratings and individual wax particles in contact. (**a**) SEM image of ommatidia of *Bemisia tabaci* surrounded by wax holding acanthae in an air-dried animal. (**b**) Cryo-SEM image of the ommatidia of *Trialeurodes vaporariorum*. (**c**) Dimple-like ommatidia gratings in an ethanol-dried animal. (**d**) Single wax particle in contact with ommatidia gratings. (**e**) Three-dimensional model of a wax particle resting on top of the mesh of ommatidia gratings. (**f**) Dimensions of hexagonal elements. Data are means ± standard deviation (N = 100 measurements).

**Figure 3:**
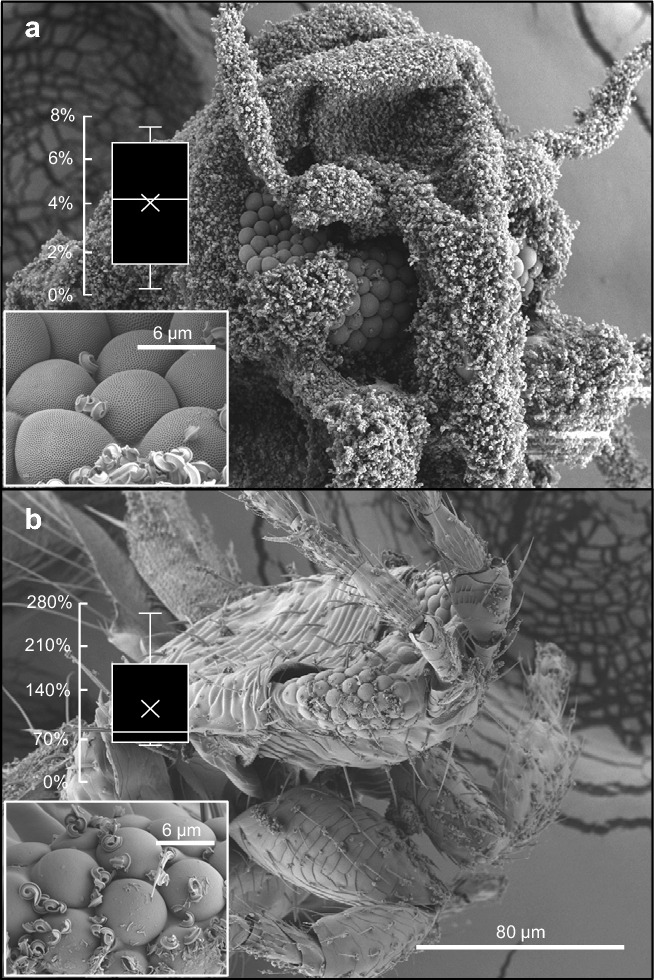
SEM images of an air dried whitefly *Bemisia tabaci* and thrip *Frankliniella occidentalis*. (**a**) Head of the whitefly is heavily covered with wax particles. Data in (**a**) and (**b**) show relative contamination of the compound eye, i.e. the ratio between number of particles on eye and body. (**b**) Head and body of the similar-sized thrips. This animal has no acanthae on its body, no dimple-like nanostructures on the cornea, and no wax canals.

Figures 2a,c and 3a show that in air-dried animals, wax particles completely cover the entire cuticle surface of the whiteflies, except for the cornea of the compound eye. Figure 2b shows that ommatidia remained clean relative to their surroundings also in Cryo-SEM images supporting that any tissue deformation during air-drying does not cause a peel-off of wax particles. We quantified wax contamination in two 40 × 30 µm^2^ areas of the cornea and the acanthae-covered area surrounding the eye and found ~ 95.8 ± 2.9% less particles on the cornea compared to the cuticle (~ 12 ± 9 vs. ~ 291 ± 78 particles, N = 5 animals, Fig. 3a). In most animals, the eye is almost free of any contamination. This strong reduction in contamination is correlated with mesh-like nanostructures on the eye surface (Fig. 2d,e) that has theoretically been considered and experimentally tested with surface replicas to be anti-adhesive, but not previously shown in an animal^17^. The nanoscale grating consists of hexagonal structures with a ~ 0.22 ± 0.007 µm diameter, ~ 30-times smaller than the cornea diameter of a single ommatidium (~ 6.6 µm, Fig. 2f). The connecting walls are 0.036 ± 0.006 µm tall and a 0.036 ± 0.006 µm thick with 0.054 ± 0.008 µm high nipples at the intersections between the walls (N = 100, Fig. 2f) leading to the castle-like features of the structure. While similar to corneal dimples, the structures here are broader and the connecting walls are thinner than previously described dimple-like ommatidia gratings of other insects^7^. The outstanding nipples, moreover, lead to a strong reduction in contact area between the cornea and wax particles. We did not observe any acanthae on or between the ommatidia of the compound eye.

The above findings put into question the mechanism which keeps the whitefly eye free of wax particles as both ommatidial grating and the absence of acanthae might be the reason for the low corneal contamination. We thus performed an additional experiment in which we investigated the similarly-sized ommatidia of the tiny thrips *Frankliniella occidentalis*. Figure 3b shows that this species has no acanthae on the outer cuticle, no ommatidial gratings nor wax canals. The thrips were kept next to the whiteflies, allowing contamination of wax particles separated from the whiteflies. Although contamination was little in the thrips, we found relative strong contamination of the compound eye, amounting to ~ 123 ± 79% (N = 5 animals, Fig. 3b) more particles on selected corneal areas than on other cuticle areas. The latter finding supports the idea that ommatidial gratings are responsible for the anti-adhesive property of the cornea and not the absence of acanthae.

## Discussion

The phenomenon of dimpled surfaces for particle attachment has previously and extensively been verified in the field of contact mechanics^36^. Nonetheless, there are no cases in which an anti-adhesive dimpled surface has been reported in an arthropod in the context of self-cleaning. Our finding of a special-shaped ommatidial grating is the first account of the existence of such a surface in an insect. The thin walls and outstanding nipples of the nanostructure are thought to strongly reduce contact area between the animal's cuticle and the extruded wax particles and lead to a significant reduction in contamination of the compound eye (Figs. 1, 2, 3). By contrast, cuticular acanthae on the body surface provide anchor points for the hydrophobic pasta-shaped particles and work diametrically, forcing wax particles to stick to the body surface making it super-hydrophobic. The properties of the two cuticular structures lead to the extreme contrast in particle accumulation seen on different body surfaces. In general, cuticle surfaces must serve multiple functions in an animal resulting from their combined mechanical and chemical properties. The cornea of the compound eye, for example, should have water and particle repelling properties, mechanical stability against abrasion, and provide antireflective properties. The prevalence of such multifunctional surfaces in nature might be low.

Many insect species, such as syrphid flies, possess ommatidia gratings in form of nano-protrusions^7^. Nano-protrusions make surfaces anti-adhesive^17^ due to the reduction of the real contact area. Dimples that result from cuticular ridges are even more effective in cases in which dimples are wide and ridges are thin^36^. Dimples of whiteflies have a strong geometric resemblance to man-made surfaces with small contact area and contact friction and adhesion^36–38^. The broad flat walls and small dimples described in bumblebees (Hymenoptera) and earwigs (Dermaptera), by contrast, do not match the latter criteria^7^. Nevertheless, mesh-like dimples similar to those in whiteflies are reported for the hydrophobic cuticular body surface of springtails^39^. A mathematical model suggests that in the springtail the mesh is formed by the reaction of substances released from excretion pores. These pores are not visible in the dimples of the whitefly ommatidia. Besides geometry, contact forces between wax particles and the nanostructured surface depend on material properties and surface chemistry^40^. As material properties of body cuticle vary up to six orders of magnitude, it is difficult to predict their significance for contact forces between ommatidia gratings and wax particles. However, we cannot completely exclude their contribution due to cuticle deformation^41^. Moreover, little is known about the conflicting tasks of corneal cuticle that must ensure both transparency and stability^42^. Surface chemistry of wax, by contrast, is comparatively well investigated in whiteflies. Wax particles consist of triacylglycerols that comprise 65–75% of the lipids in the wax^25^. Hydrocarbons account for 3–7% of the total lipids in *B. tabaci* and 0.6–1.0% in *T. vaporariorum.* Although there are distinct compositional differences between the two species, the hydrocarbons in both groups are fully saturated and contain n-alkanes and branched molecules with 18 to 40 carbon atoms^25^. Due to its hydrophobic property, wax particles easily stick to the epicuticular grease that covers the cuticle surface of most insects^43,44^. Absence or reduction of the few nanometer thick layer of grease from the cornea would contribute to the self-cleaning property of the whitefly eye.

In conclusion, this study highlights a novel nanostructure on the whitefly eye that keeps the eye free of the contaminating wax particles produced by the animal itself. The coincidence of anti-adhesive surfaces and the production of contaminants in the same animal allow us to use whiteflies as test beds for nanoscale contact mechanics and self-cleaning surfaces. In this respect, a comparative survey on nanostructured cornea in this group of insects might lead to more novel surface structures and thus blueprints for biomimetic applications.

## Materials and methods

### Animal rearing

Greenhouse whiteflies *Trialeurodes vaporariorum* Westwood, 1856 (Hemiptera, Sternorrhyncha, Aleyrodidae) are obtained from a long-time greenhouse stock culture on *Nicotiana tabacum* L. ‘White Burley’ (Solanaceae), kept in 40 × 40 × 60 cm^3^ gauze cages at 25/20 ± 2 °C (day/night), 60–70% relative humidity, and 16 h photoperiod (Institute of Phytomedicine, University of Hohenheim, Germany^45^). Silverleaf whiteflies *Bemisia tabaci* Gennadius (Hemiptera, Sternorrhyncha, Aleyrodidae) stem from stocks reared on sweet potato, eggplant and pumpkin plants, within 70 × 70 × 100 cm^3^ net growing tents. The quarantined rearing room was kept under constant environmental conditions at 27 ± 3 °C, 50%-70% relative humidity, and a 13 h photoperiod (Department of Animal Physiology, University of Rostock, Germany). We also reared thrips *Frankliniella occidentalis* that were kept under identical housing conditions *as Bemisia tabaci* and in close vicinity to the whiteflies.

### SEM image acquisition

For the Cryo-SEM, living *T. vaporariorum* were mechanically clamped in a small vice on a metal holder. The sample was subsequently frozen in a cryo-preparation chamber (Gatan, ALTO 2500 cryo-preparation system, Gatan Inc., Abingdon, UK) at 133°K. Frozen samples were sputter coated with gold–palladium (1:9, layer thickness 10 nm) in a cryo-stage preparation chamber and examined in the cryoSEM Hitachi S-4800 (Hitachi High-Technologies Co., Tokyo, Japan) at 3 kV accelerating voltage at a temperature of 153°K. Specimens of *B. tabaci* were anesthetised on a cooling plate at 1°C, placed in a fixating solution (1% paraformaldehyde, 2% glutaraldehyde, 0.1 M Phosphate buffer, pH 7.3) for one day, dehydrated twice in an ascending series of ethanol (70%, 80%, 90% and 100%, 10 min each) and critical point dried (Emitech K850, Co. Quorum Technologies LTD, East Sussex). Other *B. tabaci* and *F. occidentalis* were air dried without fixation prior to SEM analysis to preserve the particles on the cuticular surfaces. The dried specimens were then mounted on an SEM-carrier with adhesive conductive carbon tape (PLANO Co., Wetzlar, Germany), sputter coated with gold (EM SCD 004, BALTEC Co., Balzers, Liechtenstein) and imaged by field emission SEM (MERLIN® VP Compact, Co. Zeiss, Oberkochen, Germany). All data in this study are presented as means ± standard deviation.

## Data Availability

The authors declare that the data supporting the findings of this study are available within the article. Other detailed data are available from the corresponding author upon request.

## References

[1] Bernhard, C. A corneal nipple pattern in insect compound eye. *Acta Physiol. Scand.* **52**, 385–386 (1962). 10.1111/j.1748-1716.1962.tb02515.x 13971072

[2] Miskimen, G. W. & Rodriguez, N. L. Structure and functional aspects of the scotopic compound eye of the sugarcane borer moth. *J. Morphol.* **168**, 73–84 (1981). 30139179 10.1002/jmor.1051680108

[3] Fröhlich, A. A scanning electron-microscopic study of apical contacts in the eye during postembryonic development of *Drosophila melanogaster*. *Cell Tissue Res.* **303**, 117–128 (2001). 11235999 10.1007/s004410000306

[4] Stalleicken, J., Labhart, T. & Mouritsen, H. Physiological characterization of the compound eye in monarch butterflies with focus on the dorsal rim area. *J. Comp. Physiol. A* **192**, 321–331 (2006). 10.1007/s00359-005-0073-6 16317560

[5] Sukontason, K. L. *et al.* Ommatidia of blow fly, house fly, and flesh fly: Implication of their vision efficiency. *Parasitol. Res.* **103**, 123–131 (2008). 18343951 10.1007/s00436-008-0939-y

[6] Kryuchkov, M. *et al.* Analysis of micro-and nano-structures of the corneal surface of Drosophila and its mutants by atomic force microscopy and optical diffraction. *PLoS ONE* **6**, e22237 (2011). 21811578 10.1371/journal.pone.0022237 PMC3141020

[7] Blagodatski, A., Sergeev, A., Kryuchkov, M., Lopatina, Y. & Katanaev, V. L. Diverse set of Turing nanopatterns coat corneae across insect lineages. *PNAS* **112**, 10750–10755 (2015). 26307762 10.1073/pnas.1505748112 PMC4553778

[8] Turing, A. M. The chemical basis of morphogenesis. *Bull. Math. Biol.* **52**, 153–197 (1990). 2185858 10.1016/S0092-8240(05)80008-4

[9] Bernhard, C., Miller, W. H. & Móller, A. R. Function of the corneal nipples in the compound eyes of insects. *Acta Physiol. Scand.* **58**, 381–382 (1963). 14078656 10.1111/j.1748-1716.1963.tb02661.x

[10] Parker, A. R., Hegedus, Z. & Watts, R. A. Solar–absorber antireflector on the eye of an Eocene fly (45 Ma). *Proc. Roy. Soc. Lond. B* **265**, 811–815 (1998). 10.1098/rspb.1998.0364

[11] Stavenga, D. G., Foletti, S., Palasantzas, G. & Arikawa, K. Light on the moth-eye corneal nipple array of butterflies. *Proc. Roy. Soc. B* **273**, 661–667 (2006). 10.1098/rspb.2005.3369 PMC1560070 16608684

[12] Blagodatski, A. *et al.* Under-and over-water halves of Gyrinidae beetle eyes harbor different corneal nanocoatings providing adaptation to the water and air environments. *Sci. Rep.* **4**, 6004 (2014). 25103074 10.1038/srep06004 PMC5380007

[13] Huang, Y.-F. *et al.* Improved broadband and quasi-omnidirectional anti-reflection properties with biomimetic silicon nanostructures. *Nat. Nanotech.* **2**, 770–774 (2007). 10.1038/nnano.2007.389 18654429

[14] Dewan, R. *et al.* Studying nanostructured nipple arrays of moth eye facets helps to design better thin film solar cells. *Bioinsp. Biomim.* **7**, 016003 (2011). 22155981 10.1088/1748-3182/7/1/016003

[15] Palasantzas, G., De Hosson, J.T.M., Michielsen, K.L. & Stavenga, D. *Optical properties and wettability of nanostructured biomaterials: moth eyes, lotus leaves and insect wings*, 274–301 (American Scientific Publishers, 2005).

[16] Watson, G. S., Myhra, S., Cribb, B. W. & Watson, J. A. Putative functions and functional efficiency of ordered cuticular nanoarrays on insect wings. *Biophys. J.* **94**, 3352–3360 (2008). 18192379 10.1529/biophysj.107.109348 PMC2275683

[17] Peisker, H. & Gorb, S. N. Always on the bright side of life: Anti-adhesive properties of insect ommatidia grating. *J. Exp. Biol.* **213**, 3457–3462 (2010). 20889826 10.1242/jeb.043661

[18] Singer, R. & Cocucci, A. Eye attached hemipollinaria in the hawkmoth and settling moth pollination of *Habenaria* (Orchidaceae): A study on functional morphology in 5 species from subtropical South America. *Bot. Acta* **110**, 328–337 (1997). 10.1111/j.1438-8677.1997.tb00648.x

[19] Hlavac, T. Grooming systems of insects: Structure, mechanics. *Ann. Entomol. Soc. Am.* **68**, 823–826 (1975). 10.1093/aesa/68.5.823

[20] Jander, R. Grooming and pollen manipulation in bees (Apoidea): The nature and evolution of movements involving the foreleg. *Physiol. Entomol.* **1**, 179–194 (1976). 10.1111/j.1365-3032.1976.tb00960.x

[21] Schönitzer, K. & Renner, M. The function of the antenna cleaner of the honeybee (*Apis mellifica*). *Apidologie* **15**, 23–32 (1984). 10.1051/apido:19840103

[22] Szebenyi, A. L. Cleaning behaviour in *Drosophila melanogaster*. *Anim. Behav.* **17**, 641–651 (1969). 10.1016/S0003-3472(69)80006-0

[23] Rebora, M., Salerno, G., Piersanti, S., Michels, J. & Gorb, S. Structure and biomechanics of the antennal grooming mechanism in the southern green stink bug *Nezara viridula*. *J. Insect Physiol.* **112**, 57–67 (2019). 30521769 10.1016/j.jinsphys.2018.12.002

[24] Valentine, B.D. Grooming behavior in Coleoptera. *Coleopterists' Bull.* 63–73 (1973).

[25] Byrne, D. N. & Hadley, N. F. Particulate surface waxes of whiteflies: morphology, composition and waxing behaviour. *Physiol. Entomol.* **13**, 267–276 (1988). 10.1111/j.1365-3032.1988.tb00478.x

[26] Pope, R. Some aphid waxes, their form and function (Homoptera: Aphididae). *J. Nat. Hist.* **17**, 489–506 (1983). 10.1080/00222938300770431

[27] Barthlott, W. *et al.* Classification and terminology of plant epicuticular waxes. *Bot. J. Linn. Soc.* **126**, 237–260 (1998). 10.1111/j.1095-8339.1998.tb02529.x

[28] Bargel, H., Koch, K., Cerman, Z. & Neinhuis, C. Evans Review No. 3: Structure–function relationships of the plant cuticle and cuticular waxes—a smart material? *Funct. Plant Biol.* **33**, 893–910 (2006). 10.1071/FP06139 32689300

[29] Gorb, E. V. & Gorb, S. N. Anti-adhesive effects of plant wax coverage on insect attachment. *J. Exp. Bot.* **68**, 5323–5337 (2017). 28992238 10.1093/jxb/erx271

[30] Rakitov, R. & Gorb, S. N. Brochosomes protect leafhoppers (Insecta, Hemiptera, Cicadellidae) from sticky exudates. *J. R. Soc. Interface* **10**, 20130445 (2013). 23904586 10.1098/rsif.2013.0445 PMC3758005

[31] Rakitov, R. & Gorb, S. N. Brochosomal coats turn leafhopper (Insecta, Hemiptera, Cicadellidae) integument to superhydrophobic state. *Proc. Roy. Soc. B* **280**, 20122391 (2013). 10.1098/rspb.2012.2391 PMC3574307 23235705

[32] Barnes, J. & Cardoso-Vilhena, J. *Interactions between electromagnetic radiation and the plant cuticle, 170* (BIOS Scientific Publishers, 1996).

[33] Kanagaratnam, P., Hall, R. & Burges, H. Control of glasshouse whitefly, *Trialeurodes vaporariorum*, by an ‘aphid’strain of the fungus *Verticillium lecanii*. *Ann. Appl. Biol.* **100**, 213–219 (1982). 10.1111/j.1744-7348.1982.tb01933.x

[34] Samson, R. & McCoy, C. Aschersonia aleyrodis, a fungal pathogen of whitefly: I. Scanning electron microscopy of the development on the citrus whitefly. *Z. Angew. Entomol.* **96**, 380–386 (1983).

[35] Richards, A. G. & Richards, P. A. The cuticular protuberances of insects. *Int. J. Insect Morphol. Embryol.* **8**, 143–157 (1979). 10.1016/0020-7322(79)90013-8

[36] Varenberg, M., Murarash, B., Kligerman, Y. & Gorb, S. N. Geometry-controlled adhesion: revisiting the contact splitting hypothesis. *Appl. Phys. A* **103**, 933–938 (2011). 10.1007/s00339-011-6394-0

[37] Varenberg, M., Peressadko, A., Gorb, S. & Arzt, E. Effect of real contact geometry on adhesion. *Appl. Phys. Lett.* **89**, 121905 (2006). 10.1063/1.2356099

[38] Varenberg, M. & Gorb, S. N. Hexagonal surface micropattern for dry and wet friction. *Adv. Mater.* **21**, 483–486 (2009). 10.1002/adma.200802734

[39] Filippov, A., Kovalev, A. & Gorb, S. N. Numerical simulation of the pattern formation of the springtail cuticle nanostructures. *J. R. Soc. Interface* **15**, 20180217 (2018). 30089687 10.1098/rsif.2018.0217 PMC6127181

[40] Kendall, K. *Molecular adhesion and its applications: the sticky universe*, (Springer Science & Business Media, 2007).

[41] Vincent, J. F. V. & Wegst, U. G. K. Design and mechanical properties of insect cuticle. *Arthropod. Struct. Dev.* **33**, 187–199 (2004). 18089034 10.1016/j.asd.2004.05.006

[42] Li, C., Rajabi, H. & Gorb, S. N. Conflicting requirements for transparency and mechanical stability in the compound eyes of desert locusts. *Adv. Mat. Interfaces* **9**, 2200766 (2022). 10.1002/admi.202200766

[43] Voigt, D. & Gorb, S. An insect trap as habitat: cohesion-failure mechanism prevents adhesion of *Pameridea roridulae* bugs to the sticky surface of the plant *Roridula gorgonias*. *J. Exp. Biol.* **211**, 2647–2657 (2008). 18689418 10.1242/jeb.019273

[44] Voigt, D., Peisker, H. & Gorb, S. *Visualization of epicuticular grease on the covering wings in the colorado potato beetle: a scanning probe approach*, 1–16 (Springer, 2009).

[45] Voigt, D., Schrameyer, K., Kiefer, J., Zebitz, C. P. & Gorb, S. Anchoring of greenhouse whitefly eggs on different rose cultivars. *Arthropod. Plant. Interact.* **13**, 335–348 (2019). 10.1007/s11829-019-09680-5

